# Intergenerational Sharing as a Moderator of Learning and Well-being: A Late Adulthood Perspective

**DOI:** 10.1093/geroni/igaf122.3299

**Published:** 2025-12-31

**Authors:** Rong Ren, Alei Fan

**Affiliations:** Purdue University, West Lafayette, Indiana, United States; Purdue University, West Lafayette, Indiana, United States

## Abstract

This study investigates the relationships between active learning, personal growth, intergenerational sharing, and well-being among adults aged 55 and above. While lifelong learning has been associated with positive aging outcomes, the underlying mechanisms and potential moderators remain partially explored. Drawing on adult development theories and gerontological frameworks, we examined whether personal growth mediates the relationship between active learning and well-being, and whether intergenerational sharing moderates these relationships. Data were collected from 164 participants through validated measures of active learning, personal growth, intergenerational sharing, and multi-dimensional well-being. Regression analyses using PROCESS revealed that personal growth fully mediates the relationship between active learning and well-being (indirect effect = .376, 95% CI [.223, .532]), with the direct effect becoming non-significant when accounting for personal growth. Contrary to our hypothesis, intergenerational sharing did not moderate the relationship between active learning and well-being, nor the pathways in our mediation model. Instead, intergenerational sharing emerged as an independent predictor of both personal growth (β = .587, p < .001) and well-being, with its effect on well-being also fully mediated by personal growth (indirect effect = .212, 95% CI [.134, .301]). These findings suggest that both active learning and intergenerational sharing contribute to well-being through parallel pathways by fostering personal growth. The results expand our understanding of psychological mechanisms in late adulthood and suggest that interventions promoting active learning and intergenerational sharing should emphasize personal growth as a key outcome for enhancing well-being among older adults.

